# Factors associated with morphometric brain changes in cognitively normal aging

**DOI:** 10.1590/1980-57642015DN92000004

**Published:** 2015

**Authors:** Renata Eloah de Lucena Ferretti-Rebustini, Wilson Jacob-Filho, Claudia Kimie Suemoto, José Marcelo Farfel, Renata Elaine Paraiso Leite, Lea Tenenholz Grinberg, Carlos Augusto Pasqualucci, Ricardo Nitrini

**Affiliations:** 1 BSN, RN, CNS, Ph.D, Medical-Surgical Nursing Department. University of São Paulo School of Nursing. São Paulo SP, Brazil; Physiopathology on Aging Lab/ SGHC - LIM 22. University of São Paulo School of Medicine. São Paulo SP, Brazil.; 2 MD, Ph.D, Physiopathology on Aging Lab/SGHC - LIM 22. University of São Paulo School of Medicine. São Paulo SP, Brazil. Division of Geriatrics of the Clinicas Hospital. University of São Paulo School of Medicine. São Paulo SP, Brazil.; 3 BSc, Ph.D, Physiopathology on Aging Lab/ SGHC - LIM 22. University of São Paulo School of Medicine. São Paulo SP, Brazil.; 4 MD, Ph.D, Physiopathology on Aging Lab/ SGHC - LIM 22. University of São Paulo School of Medicine. São Paulo SP, Brazil; Department of Pathology. University of São Paulo School of Medicine. São Paulo SP, Brazil.; 5 MD, Ph.D, Physiopathology on Aging Lab/ SGHC - LIM 22. University of São Paulo School of Medicine. São Paulo SP, Brazil. Department of Neurology. University of São Paulo School of Medicine.

**Keywords:** aging, cephalometry, brain/anatomy & histology, health of the elderly

## Abstract

**Objective:**

Cognitive impairment is associated with reductions in brain weight and volume. The factors related to morphometric brain changes in cognitively normal aging remain unknown. We aimed to identify which clinical factors are associated with morphometric brain changes in cognitively normal aging.

**Methods:**

A cross-sectional study of 414 subjects, ≥50 years old submitted to clinical assessment and brain autopsy, after informed consent, was carried out at the São Paulo Autopsy Service, Brazil. Data on cognitive and functional evaluations were collected through structured interview applied to the next-of-kin. Brain weight (g) and volume (mL) measurements were obtained and adjusted for head circumference (cm). Associations between brain weight/volume and related factors were obtained through univariate and multivariate analysis.

**Results:**

Participants were predominantly male (60.4%), Caucasian (69%), with mean age of 67.1 ± 10.9 years. Mean brain weight was 1219.2 ± 140.9 g, and mean brain volume was 1217.1 ± 152.3 mL. Head circumference was independently associated with low brain weight (p<0.001) and volume (p<0.001). Total and adjusted brain weight and volume decreased in some conditions. Female gender (p<0.001), hypertension (p<0.009), coronary artery disease (p<0.013) and walking assistance (p<0.011) were associated with lower adjusted brain weight while schooling was associated with higher adjusted brain weight (p<0.003). Female gender (p<0.001), age (p<0.001) and hypertension (p<0.011) were associated with low adjusted brain volume.

**Conclusion:**

Morphometric brain changes occur despite the absence of cognitive impairment and were predominantly associated with age, female gender, mobility impairment and cardiovascular conditions. Schooling may be a protective factor.

## Abstract (pt)

**Objetivo:**

O comprometimento cognitivo está associado à redução de massa e volume encefálicos. Fatores associados às alterações morfométricas crânio-encefálicas durante o envelhecimento cerebral normal são escassos. Nosso objetivo foi identificar quais os fatores clínicos associados às alterações morfométricas encefálicas em indivíduos sem comprometimento cognitivo.

**Métodos:**

Estudo transversal, realizado no Serviço de Verificação de óbitos da Capital, em São Paulo – Brasil, em que 414 indivíduos, com idade ≥50 anos, foram submetidos à avaliação clínica e autópsia encefálica, após consentimento informado. A avaliação cognitiva e funcional foi obtida por meio da entrevista com familiares. Massa (g) e de volume (mL) encefálicos foram obtidos e ajustados para o perímetro cefálico (cm). A associação entre massa/volume encefálicos e os fatores relacionados (preditores) foi obtida por meio de análise univariada e multivariada. O p-valor foi fixado em 0,05.

**Resultados:**

Participantes era em sua maioria homens (60,4%), com idade média de 67,1 ± 10,9 anos, e caucasianos (69%). A média de massa encefálica da amostra foi de 1219,2 ± 140,9 g, e a média do volume foi 1217,1 ± 152,3 mL. Perímetro cefálico esteve independentemente associado à redução de massa (p<0,001) e volume (p<0,001). Massa e volume (total e corrigido) reduziu em algumas condições. Gênero feminino (p<0,001), hipertensão (p<0,009), doença arterial coronariana (p<0,013) e auxílio para deambulação (p<0,011) foram fatores associados à redução da massa encefálica corrigida, enquanto a escolaridade esteve associada com seu aumento (p<0,003). A idade (p<0,001), o gênero feminino (p<0,001) e a hipertensão (p<0,011) estiveram associados à redução no volume encefálico corrigido.

**Conclusão:**

As alterações morfométricas encefálicas ocorrem apesar da inexistência de comprometimento cognitivo e são associadas à idade, ao gênero feminino, às alterações de mobilidade e às doenças cardiovasculares. A escolaridade parece ser um fator protetor.

## INTRODUCTION

Normal and pathological brain aging has attracted great interest, largely due to the major impact of changes resulting from neurodegenerative diseases on the lives of elderly, their families and health/social support network.

Studies of morphometric brain changes have been conducted and have proven that during aging, changes in brain weight and volume occur. Controversy remains over the level at which these changes occur, due to the use of different methodological approaches. Autopsy studies and neuroimaging have shown reductions in brain weight and volume during aging.^1-14^ However, most studies did not adjust the measurements of brain weight and volume for head circumference, known to be a factor influencing brain weight and volume changes.

Some factors have been associated with brain aging^3,12,14-23^ but the role of clinical, social and demographic factors in causing morphometric brain changes remains unknown. Some studies have sought to identify factors associated with morphometric brain changes during aging, the majority of which describe the association of specific factors with cognitive impairment or neurodegenerative diseases but not with brain aging.^24-29^ Besides the identification of factors associated with dementia, it is necessary to understand factors related to normal brain aging. In 1988, Drayer^14^ suggested that the changes occurring in normal aging brains should be thoroughly studied before analyzing abnormal brains.

The objective of the present study was to identify demographic and clinical factors associated with morphometric brain changes during cognitively normal aging,

## METHODS

A cross-sectional study was conducted at the São Paulo Autopsy Service (SPAS) between 2004 and 2008. In Brazil, autopsy is mandatory for all individuals whose cause of death was not identified before death. The SPAS is a general autopsy service responsible for issuing death certificates in such cases within the city of São Paulo, Brazil.

**Participants.** Subjects were participants of the Brazilian Brain Bank of the Aging Brain Study group (BBBABSG). All the methodological procedures of the BBBABSG have been described elsewhere.^30,31^ The local Ethics Committee (CAPPesq) approved the research protocol (285/04). Briefly, participants were 50 years or older at the time of death and were referred to the SPAS for autopsy. Upon arrival of the corpses, families must come to the SPAS for funeral procedures. A team of nurses with expertise in gerontology invited a knowledgeable informant to participate in the study. A knowledgeable informant (KI) was a close family member or caregiver that has been present during the illness of the deceased and was able to recount and provide details of the deceased's health information. Subjects were included after the study procedures had been explained to the KI and they had agreed to participate by signing an Informed Consent Form (ICF).

Inclusion criteria were: subjects 50 years and older; natural cause of death (non-accidental); having a KI with close contact at least 6 months prior to death; no cognitive impairment as assessed by the Clinical Dementia Rating Scale (CDR=0). Cases with cognitive decline, no reliable informant, a medical history of advanced chronic disease or prolonged agonal state were excluded. Furthermore, only complete cases were included (those having brain weight, brain volume, head circumference and clinical data available).

**Procedures.** *Clinical Post-mortem evaluation.* Clinical evaluation consisted of assessment of the deceased's clinical and functional status in the three months prior to death. Information was obtained from the KI. A semi-structured clinical interview assessed demographics (gender, age and schooling), conditions related to death, past medical history (clinical and surgical), treatments, smoking habits and alcohol consumption, functional ability, and cognitive performance (assessed by the Clinical Dementia Rating Scale).^32^ During clinical evaluation, the interviewer continuously checked for data consistency and exclusion criteria, in order to detect any condition that might lead to the exclusion of the case. *Morphometric measurements.* At the time of autopsy, morphometric brain measurements (brain weight, volume and head circumference) were taken. Brain weight (g) was obtained by weighing the fresh brain on accurate scales upon removal from the skull. Volume (mL) was obtained by Archimedes' Principle,^33^ considered a reliable procedure for measuring volume of body regions.^34^ The principle postulates that when an object is immersed in a given liquid, the displaced liquid represents the volume of the object below the surface.^35^ Thus, the brain was immersed in water and the volume of displaced water was considered the volume of the brain.

In order to avoid the secular effect,^1,9,36,37^ brain weight and volume was adjusted for head circumference, resulting in adjusted brain weight (g/cm) and volume (mL/cm). Brain weight and volume decreases as head circumference decreases, justifying adjustment of these measures.

Head circumference (cm) was obtained by placing an inelastic tape around the skull of the corpse to obtain the maximum cranium circumference, called head circumference. For this, the tape was wrapped around the skull passing through two anatomical landmarks: the glabella and opstocranium. This measure was taken before opening of the skull and removal of the brain. In all, the procedure took about 30 minutes.

Neuropathology of the cases, to test for the existence of neurodegenerative-related pathology, was not analyzed in the present study.

**Statistical analysis.** For the descriptive analysis of sample characteristics and morphometric measures, frequency and central tendency measures were used. Brain weight and volume were associated with some clinical and socio-demographic conditions. For this evaluation, parametric and non-parametric tests were used. The t-Test (t) and Qui-square (X_2_) test were employed to check for statistical significance of continuous and nominal data, respectively. Correlations were obtained by Pearson's Correlation Test (r) for continuous data whereas Spearman's Correlation test (r) was used for ordinal data. Initially it was verified whether there was an association between morphometric variables (brain weight and volume) and each of the clinical and demographic variables. For the univariate analysis, Spearman's correlation and Kruskal-Wallis tests were used for quantitative and qualitative variables, respectively. A p-value of 0.05 was considered significant.

Using the stepwise forward procedure, multivariate linear regression selected all significant variables. Variables considered for inclusion in the regression models were those that obtained p<0.20on the Kruskal-Wallis test. Suppositions for the adjustment of the models were performed by residual analysis. Interpretation of estimated coefficients was carried out by considering the reference categories as follows: female for gender; no hypertension; no coronary artery disease and no walking assistance.

## RESULTS

Participants (n=414) were predominantly male (n=250; 60.4%), Caucasian (69.0%) and had a mean age of 67.1 ± 10.9 years. Mean age among males was 68.5 ± 11.9 years and among females was 66.2 ± 10.2 years. Mean brain weight was 1219.2 ± 140.9 g, and mean brain volume was 1217.1 ± 152.3 mL.

Adjusted brain weight and volume inversely correlated with age (r –0.291, p<0.001; r –0.259, p<0.001), meaning that both brain weight and volume reduced during the normal aging process. Adjusted brain weight and volume increased with greater years of study (r 0.239, p<0.001; r 0.166, p<0.001).

Average brain weight of individuals with previous medically diagnosed conditions was lower than for donors who had no medical condition diagnosed in life (p<0.05), irrespective of disease (Table 1). However, significant differences in average brain volume for previous medical condition in life were not found (p<0.09).

**Table 1:** Descriptive statistics for brain weight and volume (total and adjusted for head circumference), according to selected comorbidities during life.

Comorbidity	N (%)		Mean weight			Standard deviation			Mean volume			Standard deviation	
			Total (g)	Adjusted* (g/cm)		Total (g)	Adjusted* (g/cm)		Total (mL)	Adjusted* (mL/cm)		Total (mL)	Adjusted* (mL/cm)
None**	65 (15.7)		1258.3	22.5		140.1	2.2		1256.9	22.5		179.4	2.9
Stroke	47(11.4)		1191.8	21.5		150.9	3.0		1208.0	21.8		143.1	3.0
Parkinson’s	3 (0.7)		1108.3	19.5		125.7	1.2		1066.7	18.8		125.8	1.4
Depression	15 (3.6)		1186.7	21.2		98.6	1.5		1216.7	21.7		95.7	1.5
COPD	34 (8.3)		1194.9	21.3		139.6	2.3		1200.6	20.6		145.9	2.3
Hypertension	269 (65.1)		1206.9	21.4		149.3	2.5		1199.0	21.3		147.0	2.4
CAD	71 (17.2)		1211.5	21.3		144.8	2.1		1213.2	21.2		162.0	2.4
Heart failure	85 (20.6)		1214.3	21.4		133.5	1.8		1216.5	21.4		153.4	2.2
Arrhythmia	43 (10.4)		1233.9	21.7		139.4	2.5		1246.6	22.0		129.8	2.1
PVD	8 (1.9)		1212.9	21.2		135.8	1.5		1228.7	21.4		222.7	2.9
Diabetes	118 (28.6)		1207.9	21.4		130.2	1.9		1218.0	21.6		141.9	2.6
Thyroid	5 (1.2)		1201.0	21.5		92.9	1.9		1220.0	21.8		67.1	0.8
Dyslipidemia	45 (10.9)		1199.9	21.2		118.6	1.8		1212.4	21.4		142.7	2.3
Cancer	32 (7.7)		1209.5	21.8		88.1	1.5		1230.9	22.2		117.4	2.3

* Adjusted for head circumference.

** No history of diagnosed disease in life. COPD: chronic pulmonary disease; CAD: coronary artery disease; PVD: peripheral vascular disease.

The univariate analysis of data evidenced that having reported some previous diagnosis in life was statistically associated with lower brain weight (p<0.02) but not with brain volume (p<0.15). Hypertension, smoking habits and the ability to walk without any kind of assistance (walking ability) were the only variables associated with lower brain weight and volume (total and adjusted) (Table 2).

**Table 2:** P-values obtained by Kruskal-Wallis test for comparison of distribution of Brain Weight and Volume for clinical variables.

	Brain weight			Brain volume	
	Total	Adjusted		Total	Adjusted
Positive personal history of disease	**0.023**	**0.003**		0.156	0.123
Stroke	**0.057**	0.060		0.720	0.937
Parkinson’s disease	0.141	**0.044**		0.069	**0.032**
Depression	0.328	0.296		0.893	0.754
Chronic obstructive pulmonary disease	0.863	0.951		0.774	0.873
Hypertension	**0.001**	**0.000**		**0.006**	**0.001**
Coronary artery disease	0.392	**0.012**		0.739	0.180
Congestive heart failure	0.756	0.157		0.888	0.479
Arrhythmia	0.398	0.535		0.132	0.143
Vascular disease	0.939	0.478		0.455	0.655
Diabetes mellitus	0.452	0.207		0.753	0.655
Thyroid disease	0.826	0.909		0.720	0.549
Dyslipidemia	0.336	**0.090**		0.915	0.626
Cancer	0.878	0.377		0.559	0.153
Visual impairment	0.166	0.436		0.176	0.421
Hearing loss	0.735	0.906		0.628	0.328
Smoking habit	**0.000**	**0.000**		**0.002**	**0.013**
Alcohol consumption	**0.001**	**0.030**		**0.035**	0.420
Walking assistance	**0.000**	**0.000**		**0.001**	**0.005**
Physical activity practice	0.489	0.533		0.983	0.866

Head circumference, female gender, hypertension, coronary artery disease and walking assistance were associated with brain changes during aging. Schooling, understood as the number of formal years of education at school, was a protective factor against low brain weight. Regression models are shown in Table 3.

**Table 3:** Regression model for total and adjusted brain weight and volume.

**Variable**	**Brain weight**				**Adjusted brain weight**		
	**Coefficient** β	**Standard error**	**P-value**		**Coefficient ** β	**Standard error**	**P-value**
Constant	117.7	114.1	0.303		23.56	0.54	0.000
Head circumference	**21.4**	1.971	0.000		-	-	-
Gender	**57.5**	10.42	0.000		**1.03**	0.17	0.000
Age	**-1.96**	0.426	0.000		**-0.04**	0.01	0.000
Schooling	**3.4**	1.287	0.009		**0.07**	0.02	0.003
Coronary artery disease	-	-	-		**-0.53**	0.21	0.013
Hypertension	**-31.0**	9.902	0.002		**0.46**	0.18	0.009
Walking assistance	**-35.3**	13.29	0.008		**-0.60**	0.23	0.011
Bedridden state	-43.6	24.59	0.077		-0.81	0.43	0.062
**Variable**	**Brain volume**				**Adjusted brain volume**		
	**Coefficient** β	**Standard error**	**P-value**		**Coefficient** β	**Standard error**	**P-value**
Constant	116.4	161.5	0.471		**24.82**	0.74	0.000
Head circumference	**22.8**	2.8	0.000		-	-	-
Gender	**51.9**	14.6	0.000		**0.96**	0.23	0.000
Age	**-2.8**	0.6	0.000		**-0.05**	0.01	0.000
Hypertension	**-35.3**	13.8	0.011		**-0.62**	0.24	0.010

Men had 1.03 g/cm (p<0.001) higher adjusted brain weight than women. There was a 0.07 g/cm (p<0.003) increase in adjusted brain weight for each year of schooling. A 0.04g/cm reduction in adjusted brain weight was observed for each year of age (p<0.001); 0.46 g/cm (p<0.009) in the presence of hypertension; 0.53 g/cm (p<0.013) in coronary artery disease; and 0.60 g/cm if the person required assistance walking. Men also had greater adjusted brain volume than women (0.96 mL/cm; p<0.001). Adjusted brain volume decreased by 0.05 mL/cm for each year of age and in the presence of hypertension (0.62 mL/cm; p<0.010).

## DISCUSSION

The present study demonstrated that age, female gender, coronary artery disease, hypertension, and the need of assistance for walking were associated with morphometric brain changes during cognitively normal aging. Some demographic and clinical factors were associated with low or high brain weight and volume. We also demonstrated that schooling was associated with higher brain weight. According to Benedetti et al. (2006), changes in brain weight and volume are the result of a complex phenomenon caused by the association of many factors.^38^ It seems that lifestyle might play an important role in cognitive impairment. Also, it has been demonstrated that global atrophy observed in healthy subjects is lower than that observed in sick individuals.^18^

Age and gender were inversely correlated with morphometric brain measurements. Females had lower brain weight and volume when compared to men. In addition, brain weight and volume decreased with age. Previous studies have demonstrated the same association.^1,4-6,8,12,13,17,38^ The weakness of the correlations may lead to the conclusion that other factors, besides age and gender, also contribute to the phenomenon.

Formal education has been shown to be a protective factor.^39,40^ In the present study, there was an association between schooling and brain weight and volume. More years of education was associated with greater brain weight and volume.

Some risk factors have been associated with accelerated brain and volume reductions and worse cognitive performance during aging, such as hypertension, diabetes, dyslipidemia, alcohol consumption, smoking habits, obesity, physical inactivity and elevated plasma homocysteine level.^15,16,18,20-22,27,42,43^ In the present series, stroke, Parkinson's disease, hypertension, coronary artery disease, smoking habits, alcohol consumption and walking assistance were positively associated with lower brain weight or volume, but only coronary artery disease and hypertension remained in the final regression model.

The results of the multivariate analysis lead to a better understanding of the role of each factor associated with brain weight and volume changes. Age, female gender and hypertension were correlated with lower brain weight and volume in the total and adjusted regression models.

Head circumference also influenced brain weight and volume, increasing these parameters by 2.4 g and 2.8 mL, respectively, for each additional centimeter of circumference. Despite the observation of an increase in brain weight and volume with increased years of schooling, the correlation was verified only for brain weight but not for volume. Some subjects may exhibit superior functioning even in the presence of neuropathology. This is attributed to the so-called reserve.^39^ Cognitive reserve probably implies brain reserve,^40,41^ related to brain mass. Further studies correlating the interactions between schooling and volume might allow more conclusive interpretations.

Heart failure, described elsewhere as responsible for brain changes,^16-19,24^ was not associated with brain weight or volume in the present study. Likewise, depression did not correlate with morphometric brain changes, in contrast to previous studies which have demonstrated this interaction.^44^ This disparity is probably because the detection of Depression in the present sample, as a medical condition diagnosed in life, was based on a collateral source while the overall number of subjects with established depression diagnosis in life was small. Diabetes has been described as an important factor related to brain weight and volume^18^ but this association was not found in the present study. This absence of association also occurred for physical activity, which may be explained by the sample profile of sedentary elderly subjects.

Cardiovascular risk factors have been implicated for increased mortality rates and morbidity among elderly,^45,46^ especially when in association with psychiatric morbidity. Many factors can contribute to brain weight in subjects with cardiovascular disease. Cardiovascular risk factors are associated with strokes and white matter lesions, which might contribute to cognitive decline, depending on their location, type and number.^47^ Atrial fibrillation, heart failure and hypertension may lead to reduced blood flow to the brain with decreased tissue perfusion or bleeding. This may contribute to a decrease in supply of oxygen and other nutrients to the brain, resulting in cognitive impairment and brain atrophy.^16,47^ Nevertheless, not all the cardiovascular risk factors investigated in the present study were selected for inclusion in the regression model. Hypertension alone, was responsible for a 34g reduction in brain weight.

The strength of this study is the clinicopathological assessment of the subjects in a post-mortem setting, which allowed direct brain morphometric measurements in an adequate number of cases. The frequency of autopsies is decreasing worldwide. In Brazil, autopsy procedures are mandatory for all subjects who die without a defined medical condition. Consequently, the number of cases submitted to autopsy every year is huge.

Another strength of the present study is the sample comprising cognitively normal subjects. This characteristic is important because all the findings of the study relate to a sample comprising subjects without cognitive impairment. Morphometric brain changes were present during aging but were not sufficient to cause cognitive impairment or dementia. The primary hypothesis is that, as occurs in other organisms, there is a brain reserve (cerebral reserve) capable of maintaining cognitive brain function under normal conditions. Brain aging involving isolated morphometric changes is not associated with cognitive impairment.

One of the study limitations is the fact that the presence of neuropathological lesions, such as neurofibrillary tangles, senile plaques, infarcts, or synucleinopathies, which can explain brain atrophy, were not analyzed. Neuropathological lesions may play an important role in reducing brain weight and volume. The contribution of these lesions to morphometric changes, associated with other changes, could not be determined due to the lack of neuropathology.

Another limitation was that cross-sectional studies are not sufficient to establish causality, but can reveal associations. Longitudinal studies, with brain donation in life, could contribute to a better understanding of the process. Additionally, the postmortem assessment of cognitive function and the fact that informants reported all medical conditions can also be considered a limitation.

Finally, the results of this study are important to public health. Since it is known that some diseases and life-styles are associated with morphometric brain changes during aging and that these changes can reduce brain reserve and contribute to cognitive decline, efforts have to be made toward preventing these conditions and reinforcing public health measures, related to health promotion.
